# Gas-Sensing Study and Applications of Triboelectric Nanogenerator-Powered CuO-Modified CeO_2_ Nanomaterials for Ammonia Sensor at Room Temperature

**DOI:** 10.3390/s25092753

**Published:** 2025-04-26

**Authors:** Junsheng Ding, Yingang Gui, Hua Huang

**Affiliations:** College of Engineering and Technology, Southwest University, Chongqing 400715, China; djsdjs123@email.swu.edu.cn (J.D.); yinganggui@swu.edu.cn (Y.G.)

**Keywords:** NH_3_ gas sensor, heterojunction, nanogenerator, wide detection range

## Abstract

Ammonia (NH_3_) is a common agricultural gas, and its accurate detection is critical to agricultural production. In this study, nano-CuO/CeO_2_ composites were prepared to achieve a wide range of ammonia detection at room temperature. Characterization data verified the composite heterojunction structure of CuO/CeO_2_, which demonstrates an outstanding large specific surface area for ammonia detection. It provides more active sites for NH_3_ molecules, which brings a very high response to ammonia (70.3% @100 ppm NH_3_), a large detection range (0.5–200 ppm NH_3_), and a fast response/recovery time (13 s/17 s @20 ppm NH_3_). Systematic testing showed that the nano-CuO/CeO_2_ composites also exhibit excellent extended-term stability and selectivity. Further studies showed that the p-n heterojunction structure of CuO/CeO_2_ allowed the composite to retain its gas-sensitive properties to ammonia, in addition to the improved ammonia-detection range of the composite based on the synergistic effect of these two materials. The mechanism of CuO/CeO_2_ heterojunction nanocomposites towards ammonia detection was also elucidated from a microscopic perspective at the molecular level. Finally, a triboelectric nanogenerator (TENG) that can be driven by wind power has been prepared, upon which the feasibility of the combination of the TENG and the ammonia sensor to realize environmental monitoring was investigated.

## 1. Introduction

As the two pillars of modern agriculture, farming and cultivation are related to the economic development of the country and the quality of people’s daily diet [1,2,3]. In the process of crop growth, the use of chemical fertilizers produces a variety of pollutant gases [4,5,6], which have a bad impact on the environment, crop growth, and poultry development [7,8,9]. Ammonia, as a pollutant gas, is widely present in all aspects of agricultural production [10,11,12,13]. Most countries have strict control standards for ammonia concentrations in farming and cultivation [14,15]. For example, the maximum time people can be exposed to ammonia at 25 ppm should not exceed 8 h, and at 35 ppm, it should not exceed 10 min [16]; in farming, the maximum concentration of ammonia in pigsties should be controlled between 25 and 50 ppm, and if the concentration exceeds 50 ppm, it will have a negative impact on the health of pigs [17]. Therefore, it is particularly important to detect ammonia at room temperature in agricultural processes, in farming, and in human safety monitoring.

Cerium oxide is an n-type semiconductor with confirmed physical and chemical stability, corrosion resistance, and non-toxicity, and it has proven to be an excellent metal oxide gas-sensing nanomaterial for environmental detection [18,19,20]. For example, Sipra Choudhury et al. explored a CeO_2_ film using NO_2_ gas sensing at the ppm level [21], and Tokeer Ahmad et al. prepared ZrO_2_/CeO_2_ composites for the detection of CO [22]. According to a recent study by Zhang Dongzhi et al. [14], CeO_2_ can achieve an extremely low concentration of NH_3_ (36.8% @50 ppb NH_3_) at room temperature. However, their study also showed that a CeO_2_-built NH_3_ material exhibits a lower gas sensing limit value (lower to 10 ppm). Therefore, the NH_3_ sensor based on pure CeO_2_ is not suitable for ammonia detection in agricultural processes, so it is necessary to further extend its detection range. In addition, the response/recovery time of the pure CeO_2_-built NH_3_ sensor has to be further improved to achieve wide-range and fast NH_3_ detection.

Compounding with other materials is an impactful measure to expand the NH_3_-detection range of CeO_2_ gas-sensitive sensors [23]. For example, Zheng Cai et al. prepared a SiO_2_/CeO_2_ gas-sensitive material, which realized high-performance ammonia sensing [24]; Vikas B. Patil et al. prepared a PANI/CeO_2_ gas-sensitive sensor and realized ammonia detection at room temperature [25]. Although the sensors based on the above composite materials have expanded the ammonia-detection range in some way, their optimal operating temperatures, response values, and response/recovery times have been affected to varying degrees. Therefore, it is necessary to find a material to minimize the adverse effects of material doping. The study shows that the construction of P-N junctions will notably shorten the response/recovery time and expand the gas-sensing range of the material [26,27,28,29]. Furthermore, the particle size and shape of composite materials affect the sensing performance by influencing the specific surface area. Sanju Gupta et al. investigated the impact of the morphology and topological network assembly of zinc oxide (ZnO) nanostructures, finding that nanorods and tetrapod arms exhibit superior sensing performance [30]. This provides insights for material preparation. CuO is a typical P-type semiconductor, and its structural stability and simplicity of preparation have been widely used in the multi-material composite [31,32,33]; for example, Li Li et al. realized highly differentiated NH_3_ and H_2_S gas sensing using an In_2_O_3_@CuO multijunction nanofiber-built gas sensor [34]; Fei Wang et al. utilized CuO/WS_2_ nanohybrids for the fast and efficient detection of NH_3_ at room temperature [35]; Yamahara, Hiroyasu et al. designed an SnO_2_-CuO heterostructured nanofiber with enhanced NH_3_ gas-sensing performance [36]. The above studies show that CuO has a wide detection range for ammonia. Therefore, the modification of CeO_2_ with CuO is expected to obtain an ammonia sensor with a fast response/recovery time and wide detection range.

In addition, with the development of the Internet of Things (IoT), agricultural production is evolving in the direction of intelligentization [37]. A large number of sensors are used for monitoring various environmental parameters, which largely increases the cost of laying and maintaining sensor lines. Over the past few years, the triboelectric nanogenerator has been regarded as an effective way of harvesting environmental energy with a simple structure, facile preparation, and easy integration, which has received much attention from researchers [38,39,40]. Taotao Zhan et al. proposed a portable self-powered intelligent fluid pressure sensor based on a liquid piston triboelectric nanogenerator (LP-TENG) [41]. There is much energy in the environment and agricultural greenhouses that we fail to utilize, such as wind energy in air and from exhaust fans. Therefore, rationally designing the structure of rotary triboelectric nanogenerators or enhancing their output through ion implantation and charge stimulation holds promise for providing viable solutions to energy harvesting from the environment, which could support the deployment of sensors in smart agriculture [42].

Here, CeO_2_ and CuO nanoparticles were synthesized using calcination and hydrothermal methods, and CuO/CeO_2_ composites were obtained via ultrasonic compounding and calcination. The micro-morphology was investigated by means of characterization, such as SEM, EDS, XRD, and TEM, to verify the structure of the repaired CuO/CeO_2_ composites. The gas-sensing performance, response/recovery time, cyclic stability, selectivity, and extended-term stability of CeO_2_ and CuO/CeO_2_ composite sensors for ammonia were detected under multiple gas concentration conditions. The results demonstrate that the composite of CuO successfully extends the detection range and improves the response/recovery performance of CeO_2_ sensors for ammonia. Finally, the reasons for the combined impact of CuO/CeO_2_ composites and the improvement of the p-n heterojunction on ammonia gas sensing performance were further investigated. Finally, the feasibility of combining the triboelectric nanogenerator and ammonia sensor for environmental monitoring is investigated.

## 2. Materials and Methods

### 2.1. Materials

Cerium nitrate hexahydrate, anhydrous ethanol, copper acetate, and sodium hydroxide were bought from Aladdin Ltd. (Shanghai, China). All materials were not further purified.

### 2.2. Preparation of Composite Materials

**Figure 1:**
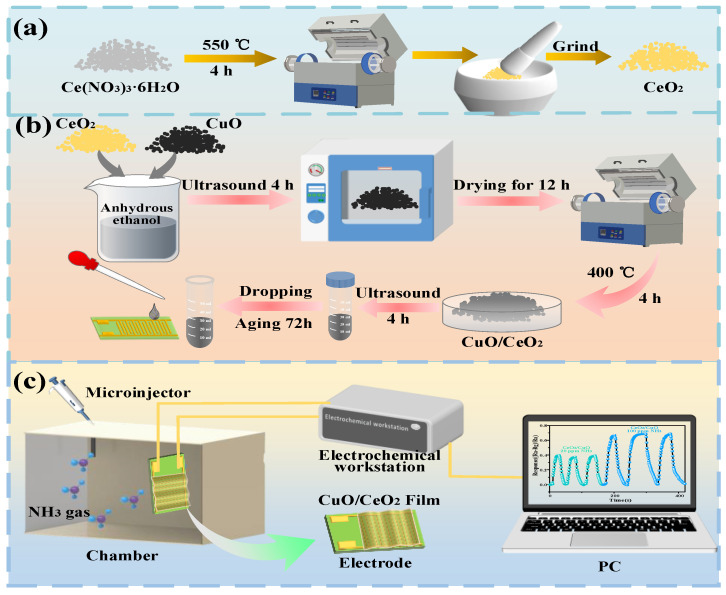
Preparation of materials. (**a**) Preparation process of CeO_2_, (**b**) preparation of composite material and gas-sensitive sensor, and (**c**) gas-sensitive testing process.

Firstly, CeO_2_ nanoparticles were prepared by calcining cerium nitrate hexahydrate at 550 °C for 4 h to prepare the CeO_2_ nanomaterial. The prepared CeO_2_ was put into the milling bowl and milled for spare, and the preparation process is shown in Figure 1a.

Nano-CuO was prepared using the precipitation method; 2.0 g of copper acetate was mixed in 80 mL of anhydrous ethanol and stirred for 30 min, and then, 0.8 g of sodium hydroxide was added. The mixed solution was then moved to a Teflon-lined stainless steel autoclave, sealed, and kept at 120 °C for 2 h. After spontaneous cooling down naturally, the black Nano-CuO precipitate was extracted via a centrifugation process and washed several times with anhydrous ethanol and pure water. Eventually, the Nano-CuO was dried in air at 60 °C for 24 h.

Finally, the preparation of the composite materials, CeO_2_, and CuO were added to anhydrous ethanol according to different molar ratios (1:1, 2:1, 3:1). Then, the composites were ultrasonicated for 4 h to make a uniform composite and then placed in an oven for drying for 12 h. Eventually, the composites were calcined at 400 °C for 4 h to obtain CuO/CeO_2_ composites with different composite ratios. The preparation process is shown in Figure 1b. The optimal response to NH_3_ was observed when the molar ratio of CeO_2_ and CuO was 1:1. Under this condition, SEM and other methods were used to investigate the microstructure of the mixed materials and conduct experiments.

### 2.3. Preparation of Gas-Sensitive Sensors

The prepared CuO/CeO_2_ composite was added at 10 mg to an appropriate amount of anhydrous ethanol and magnetically stirred for 5 h to obtain a well-mixed solution. Then, the mixed solution was added dropwise onto the fork finger electrode. Finally, the gas-sensitive materials were dried at 60 °C for 24 h to obtain a CuO/CeO_2_ composite-based gas sensor. The preparation process is shown in Figure 1b.

### 2.4. NH_3_-Sensing and Measuring Device

The entire procedure for gas-sensing measurement adopts the static gas distribution method. The experiment was conducted under room temperature conditions (30 °C, 50% RH). The injection liquid volume into the gas-sensing chamber was quantified using Equation (1).

(1) $$Q=\frac{V\times c\times M}{22.4\times d\times p}\times 10^{-9},$$

*Q* (mL) represents the quantity of the injected liquid. *V* (mL) shows the internal space size of the gas-sensing chamber. *c* (ppm) denotes the concentration value of the tested gas object. In addition, *M* (g/mol), *d*, and *p* (g/cm^3^) represent the relative molecular mass, purity, and density of the liquid, respectively. The test process is as presented in Figure 1c. The prepared gas sensor was moved from the air-filled atmosphere to the pre-made ammonia-containing atmosphere. This process continued until the resistance value of the sensor became stable. After that, the sensor was relocated back to the air environment. The resistance variation was measured using an electrochemical workstation, and the average value was used after repeating the above gas-sensing procedure five times to be used for the calculation of the gas response. The sensor’s response (S) was calculated using the formula S = (R_a_−R_g_)/R_a_, where R_a_ represents the steady-state resistance of the sensor in an air environment, while R_g_ stands for the mean resistance of the sensor when exposed to a particular concentration of NH_3_. The response/recovery time of the CuO/CeO_2_ composite built sensor is calculated once the gas-sensing value reaches 90% of total response value.

## 3. Results and Discussion

### 3.1. Characterization Results

**Figure 2:**
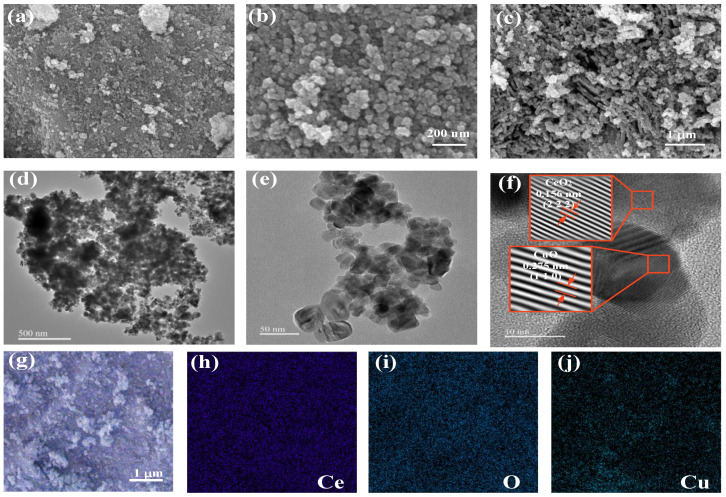
Characterization of the materials. (**a**,**b**) SEM characterization of CeO_2_ at different scales, (**c**) SEM characterization of CuO, (**d**–**f**) TEM images at different scales, (**d**) 500 nm, (**e**) 50 nm, (**f**) 10 nm. (**g**) Three-element composite images of the composite Ce, O, and Cu, and (**h**–**j**) elemental characterization images of Ce, O, and Cu, respectively.

In order to demonstrate the successful synthesis of CeO_2_ and CuO nanoparticles, the prepared materials were characterized via SEM. The SEM characterization of CeO_2_ at different scales is shown in Figure 2a,b, the fabricated CeO_2_ reveals a uniform nanoparticle shape. Figure 2c demonstrates the prepared CuO nanoparticles, and it can be seen that the CuO nanoparticles were successfully prepared with a size of about 50 nm. The prepared CeO_2_ exhibits a porous structure, with CuO particles measuring 50 nanometers, indicating that CuO can adhere more uniformly to CeO_2_, thereby increasing the specific surface area. The microstructure of the composites was then morphologically characterized via TEM analysis. Figure 2d–f demonstrate the TEM results with different magnification ratios, which verify the successful synthesis of CeO_2_ and CuO nanoparticles. Figure 2d,e illustrate that in the composite material, CuO is uniformly combined on CeO_2_, with no noticeable large agglomerated areas, indicating that the composite material has a large specific surface area. Figure 2f demonstrates the lattice spacing of the two materials, where 0.156 nm corresponds to the (222) crystal plane of CeO_2_ and 0.275 nm corresponds to the (110) crystal plane of CuO. A clear interface is displayed between the two, indicating that the two materials form a good contact and a heterojunction, which is conducive to obtaining better gas sensitization. To verify the successful synthesis of CuO/CeO_2_ composites, the prepared CuO/CeO_2_ composites were characterized via EDS, and the test results are presented in Figure 2g–j. Figure 2g demonstrates the distribution images of Ce, O, and Cu elements together. The Ce elements demonstrated in Figure 2h are from CeO_2_. The O elements in Figure 2i are from CeO_2_ and CuO. The Cu elements in Figure 2j are totally from CuO, and the uniform distribution of its EDS image also indicates that CuO is highly dispersed on the CeO_2_ support, which is conducive to increasing the specific surface area. These verify the successful synthesis of the CuO/CeO_2_ composites.

The physical phases of CeO_2_, CuO, and CuO/CeO_2_ were received via XRD tests, and all samples were scanned within a diffraction angle of 20–80°. The scanning data are shown in Supplementary Materials, Figure S1, and the characteristic peaks of CeO_2_ are located at 28.55°, 33.08°, 47.48°, 56.34°, 59.09°, 69.41°, 76.70°, and 79.077°, corresponding to the (111), (200), (220), (311), (222), (400), (331), and (420) facets, respectively. The CuO has more diffraction peaks, among which 35.42° and 38.71° correspond to the (002) and (111) crystal planes, respectively. In the XRD curves, the characteristic peaks of the CuO/CeO_2_ composite material include the characteristic peaks of both CuO and CeO_2_. In Supplementary Materials, Figure S2, we obtained the XRD curves of the product standard by querying the card number (Reference code of CuO: 00-045-0937, Reference code of CeO_2_: 00-034-0394). Comparing Figure S1, we found that the peak positions of the two were almost identical, and the relative size relationship of the peaks also matched, which proves the successful synthesis of the CuO/CeO_2_ composites. In addition, the XRD profiles are all sharper, indicating that the prepared samples have good crystallinity.

### 3.2. Ammonia Gas-Sensing Performance of CuO/CeO_2_ Sensor

Connect a pair of electrodes to CuO/CeO_2_ thin films and place them in a gas chamber filled with ammonia. The change in the resistance of the CuO/CeO_2_ is measured using the electrodes on an electrochemical workstation. When the resistance stabilizes at a certain concentration, we remove the CuO/CeO_2_ thin films to the air and change the concentration for the next measurement.

**Figure 3:**
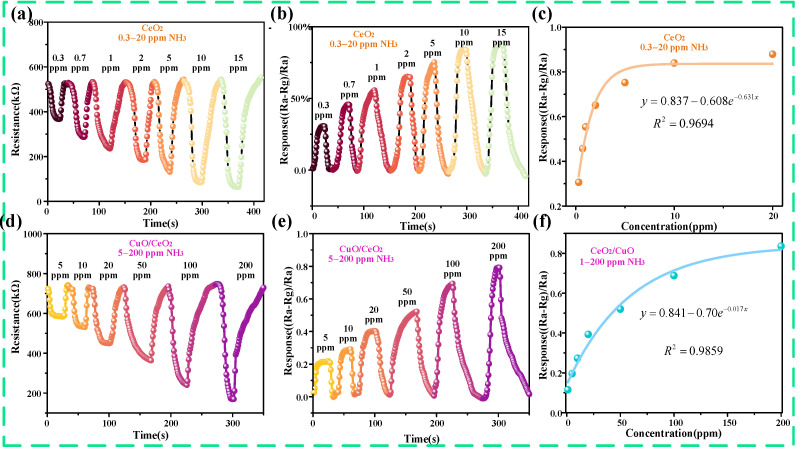
Gas-sensitive properties of the materials. (**a**) Resistive response of CeO_2_ to different concentrations of ammonia, (**b**) response values of CeO_2_ to different concentrations of ammonia, (**c**) fitted curves of response values of CeO_2_ to different concentrations of ammonia, (**d**) resistive response of CuO/CeO_2_ to different concentrations of ammonia, (**e**) response values of CuO/CeO_2_ to different concentrations of ammonia, and (**f**) fitted curves of response values of CuO/CeO_2_ to different concentrations of ammonia.

First, the gas-sensing properties of the CeO_2_ gas sensor and CuO/CeO_2_ gas sensor were detected under multiple gas concentrations conditions: 0.3 to 20 ppm, and the resistive responses of the CeO_2_ gas sensor and CuO/CeO_2_ gas sensor are shown in Figure 3a,d. From the two figures, the stabilized resistances of the CeO_2_ sensor and CuO/CeO_2_ sensor in air were 520 kΩ and 720 kΩ. Due to the interaction of CeO_2_ and CuO/CeO_2_ with NH_3_ molecules, the resistance of these sensors exhibited a downward trend as the ammonia concentration increased. From Figure 3b,e, it can be seen that the CeO_2_ sensor has excellent gas sensitivity for ammonia, with a very good response at very low ammonia concentrations. At a 0.3 ppm ammonia concentration, the response value is up to 0.31. The corresponding response of the CuO/CeO_2_ sensor at 5 ppm is 0.21, which indicates that it can also realize the detection of ammonia gas at lower concentrations. Furthermore, as the ammonia concentration increases, the response times of both CeO_2_ and CuO/CeO_2_ decrease. The fitted curves of response values for CeO_2_ sensors at a 0.3–20 ppm ammonia concentration are shown in Figure 3c. The fitting function is y = 0.837 − 0.68e^−0.631x^. From the fitted curves of the response values, the sensor responds well to ammonia in the concentration range of 0.3–10 ppm, and there is a clear difference in the response values. The slope of the curve is zero after 10 ppm, indicating that once the ammonia concentration exceeds 10 ppm, the resistance of the CeO_2_ sensor does not change with varying gas concentrations, resulting in a response plateau. However, the response value of the sensor hardly changed with a further increase in the ammonia concentration, which indicates that the CeO_2_ sensor has a low upper limit for NH_3_ detection, largely limiting its application. The fitted curves of the response of the CuO/CeO_2_ sensor for different concentrations of ammonia are shown in Figure 3f, and the fitting function is y = 0.841 − 0.7e^−0.017x^. According to the fitting results, the lower limit of detection can be up to 0.5 ppm, which indicates that the composite sensors prepared still have a lower limit of detection. As the ammonia concentration increases, the curve becomes increasingly flat after 100 ppm, indicating that the resistance change of the CuO/CeO_2_ sensor is very subtle. Particularly at 200 ppm, the curve slope is almost zero, at which point a response plateau is observed. In addition, and most importantly, the sensor’s gas-sensing response to high concentrations of ammonia gas was further improved with the composite of CuO, and the sensor was able to detect ammonia very well in the range of 5–200 ppm; this indicates that the modification of CuO introduces additional active sites for NH_3_ absorption attributed to the heterojunction-induced electronic modulation and surface defect engineering.

**Figure 4:**
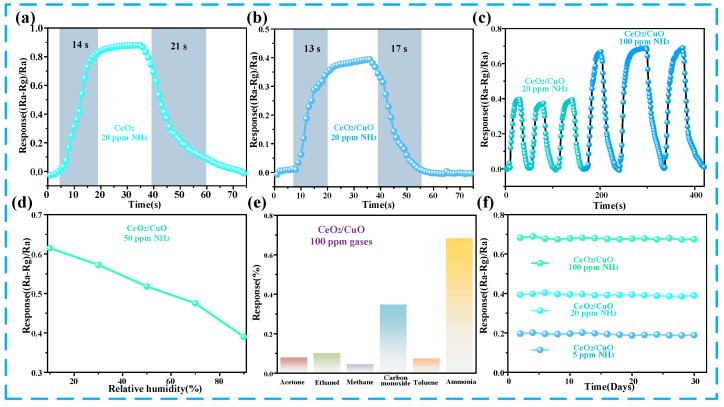
Performance study of CuO/CeO_2_ sensor. (**a**,**b**) Sensor response/recovery time at 200 ppm and 20 ppm ammonia concentrations, (**c**) three-cycle test of the sensor at 20 ppm and 100 ppm ammonia concentrations, (**d**) variation in the sensor with humidity, (**e**) selectivity of the sensor to common gases, and (**f**) extended-term stability of the sensor.

Different parameters of the prepared sensors were measured in order to verify that the prepared sensors have excellent performance. Firstly, the response/recovery time of the CeO_2_ sensor and CuO/CeO_2_ sensor were tested in an ammonia atmosphere at 20 ppm, as shown in Figure 4a,b. Also, CeO_2_ and CuO/CeO_2_ sensors have a fast response/recovery time for ammonia, and the response/recovery time of the CuO/CeO_2_ sensor is 13 s/17 s at 20 ppm, which is lower than that of CeO_2_ sensor. Figure 4c represents the cyclability of the prepared CuO/CeO_2_ sensors, which showed good cyclability at 20 ppm and 100 ppm ammonia concentrations, and their response remained essentially unchanged at the same concentration. In Figure 4d, the response of the CuO/CeO_2_ sensor to 50 ppm ammonia gas at 20–90% humidity was tested. The response value of the sensor displays a downward trend with an increasing humidity, because H_2_O molecules also interact the active sites on the surface of the CuO/CeO_2_ composites under high-humidity conditions, resulting in less ammonia adsorption and a low gas-sensing response value. The selectivity of the sensor is shown in Figure 4e. Here, the cross interferences of five common interfering gases (acetone, ethanol, methane, carbon monoxide toluene) were detected. The CuO/CeO_2_ sensor has the largest response to ammonia, which indicates that the CuO/CeO_2_ sensor has excellent selectivity to ammonia. Finally, the extended-term stability is also a crucial indicator to determine whether the sensor can be used for a long time or not. Figure 4f tested the response of the composite sensor under different ammonia concentrations for 30 days, and the response of the sensor remained stable, which proved that the outstanding combination with CuO/CeO_2_ and NH_3_ molecules.

### 3.3. Mechanism of the CuO/CeO_2_ Sensor

**Figure 5:**
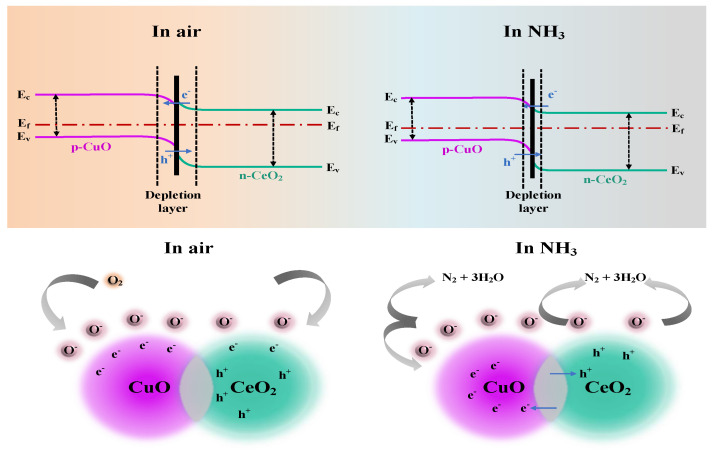
Gas-monitoring mechanism.

The ammonia gas-sensing mechanism of the CuO/CeO_2_ sensor is schematically presented in Figure 5. The gas-sensing response of the sensor depends on the active sites offered by the CuO/CeO_2_ heterojunction nanomorphology on one hand and is determined by the resistance modulation ability on the other hand. In air, the energy bands at the interface bend due to the different work functions when p-type CuO is in contact with n-type CeO_2_ and eventually reach the same Fermi energy level. Simultaneously, the electrons will shift from the conduction band of CeO_2_ to form p-n junctions at the CuO/CeO_2_ heterojunction interface [43]. When the sensor is exposed to air at room temperature, oxygen molecules (O_2_) interact with the surface of the gas-sensitive materials. These adsorbed O_2_ molecules can capture free electrons from CuO/CeO_2_ to form oxygen ions (O_2_^−^) through the following reaction, as shown in Equations (2) and (3) [44]. This process leads to a more pronounced immobilization of electrons within the conduction band. As a consequence, the width of the electron-depletion layer expands further, causing an elevation in resistance. On the contrary, when the sensor is exposed to an ammonia gas, NH_3_ molecules will interact with the active sites of the sensitive materials. Then, the adsorbed NH_3_ molecules react with the pre-adsorbed O_2_^−^ as shown in Equations (4) and (5) [45].

(2) $$O_{2}(gas)↔O_{2}(ads),$$

(3) $$O_{2}\left(\begin{array}{c} ads \end{array}\right)+e^{-}\to O_{2}^{-}\left(\begin{array}{c} ads \end{array}\right),$$

(4) $$NH_{3(\mathrm{gas})}\to NH_{3(\mathrm{ads})},$$

(5) $$NH_{3(\mathrm{ads})}+3O_{2(\mathrm{ads})}^{-}\to 2N_{2}+6H_{2}O+3e^{-}$$

The trapped electrons are released back into the conduction band through this process. This action boosts the concentration of free electrons. As a result, the depletion layer becomes thinner, leading to a decrease in resistance and thereby enabling resistive modulation.

The enhanced gas-sensing performance of the sensor towards ammonia is mainly due to the synergistic effect of the two materials. On the one hand, CeO_2_ is an n-type semiconductor material used for NH_3_ detection at the ppb level. CeO_2_ exhibits a remarkably high response to NH_3_ but has a narrow detection range (upper detection limit of 10 ppm). On the other hand, CuO is a p-type semiconductor material with a large detection range for NH_3_, but it has a low response value towards NH_3_. By combining the two materials, CuO can contribute to the more effective adsorption of NH_3_ in the composite materials, while CeO_2_ can contribute to the better oxidation of NH_3_ in the composite materials, which enables the obtained materials to have a wide detection range and a high gas-sensitive response for the detection of NH_3_.

### 3.4. Application

**Figure 6:**
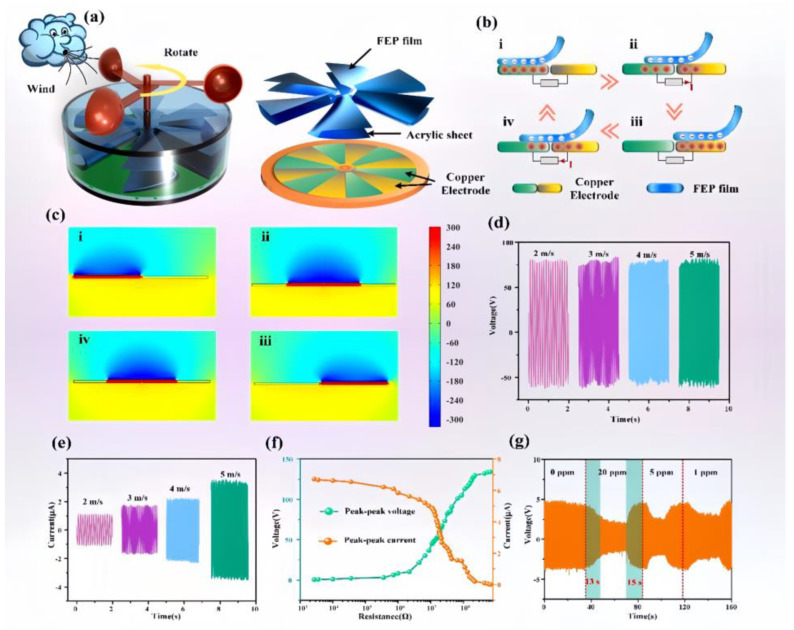
(**a**) Structural diagram of the prepared rotary TENG and schematic diagrams of its rotor and stator, (**b**) the working principle of the TENG, (**c**) finite element simulation analysis of the TENG, (**d**) the open-circuit voltage and (**e**) the short-circuit current of the TENG at different wind speeds, (**f**) the impedance matching curve of the TENG at a wind speed of 5 m/s, and (**g**) the voltage variation curve of the TENG-powered NH_3_ sensor in response to changes in the NH_3_ concentration.

There is usually energy generated during agricultural production that fails to be recycled, such as wind energy from exhaust fans in farming and smart farms. The exhaust fan is also the most responsive part to the gas concentration in the greenhouse or farm. Based on this, a rotating TENG has been fabricated in this work, which is used to harvest wind energy from the environment and then drive the gas sensor to operate. In order to harvest wind energy from the environment as much as possible and to reduce the activation wind speed, as well as the manufacturing cost, a TENG structure with low friction resistance is fabricated, as displayed in Figure 6a. The triboelectric nanogenerator mainly consists of an FEP dielectric layer, bottom copper electrode, and driving wind cup. According to the previous research of our group, the dielectric layer with a flag type features the optimal output [2]. The working principle of the TENG is presented in Figure 6b. In the initial state, the FEP film is completely overlapped with the copper electrode, and a negative charge will be generated on the electro-negative FEP film due to electrostatic induction and the friction-charging effect. Based on the principle of charge conservation, an equivalent quantity of positive charges is produced on the relevant copper electrode. As the FEP membrane moves rightward, to counteract the electric field brought about by the FEP membrane, the positive charge on the left electrode migrates to the adjacent electrode via the external circuit, consequently generating an electric current. Once the FEP membrane has fully shifted to the adjacent electrode, all the positive charges are transferred to that very electrode. This process will be repeated as the rotor turns, thus producing a constant AC output. Figure 6c illustrates the simulation results of the TENG correspondingly, which confirms the voltage-output capability of the TENG. Figure 6d,e demonstrate the open-circuit voltage and short-circuit current of the prepared triboelectric nanogenerator under different wind speeds. The designed TENG exhibits a low turn-on wind speed. With the wind speed increasing, the open-circuit voltage of the TENG basically remains stable, while its short-circuit current is positively correlated with the wind speed. This is due to the fact that the open-circuit voltage of the TENG is mainly affected by the effective contact area, while the effective contact area of the rotating TENG remains basically constant during operation, so its open-circuit voltage remains basically stable. However, for the short-circuit current, the increase in the driving wind speed leads to an increase in the rotational speed of the TENG, which increases its charge-transfer rate, so the current increases with the increasing wind speed. The stabilized output voltage facilitates the construction of the subsequent sensing system. Figure 6f presents the impedance matching curve of the TENG. Its internal resistance is around 23 MΩ. Figure 6g exhibits the voltage-change curves on the sensor sides when the TENG drives the NH_3_ sensor to work. Under a constant wind speed condition, changing the ammonia concentration does not alter the TENG’s output power. As the ammonia concentration in the environment increases, according to Figure 3d, the sensor resistance decreases, leading to a reduction in the output voltage to maintain constant power. In Figure 6g, the output voltage is the highest when the gas is air, corresponding to the maximum resistance in Figure 3d. Under 1 ppm, 5 ppm, and 20 ppm ammonia concentrations, the output voltage decreases, which aligns with the resistance change pattern. This verifies the feasibility of using triboelectric nanogenerators for the self-powered detection of different NH_3_ concentrations.

## 4. Conclusions

In summary, CuO and CeO_2_ nanoparticles were successfully synthesized and CuO/CeO_2_ composites were prepared via ultrasonic compounding and calcination. The materials were fully characterized to demonstrate the successful preparation of the composites and the preparation of CuO/CeO_2_ sensors for ammonia gas sensing. Experiments were conducted under 50% air humidity conditions, and the results indicated that CuO/CeO_2_ heterojunction nanocomposites exhibit a broader NH_3_-detection range and accelerated response/recovery kinetics, attributed to molecular-level interfacial charge transfer and optimized band alignment induced through heterojunction engineering. The results show that CuO/CeO_2_ is an excellent and ideal material for wide-range ammonia detection at room temperature, and we also explained the mechanism of the detection process from the molecular side, concluding that the ammonia-sensing mechanism of the CuO/CeO_2_ sensor is based on the heterojunction interface characteristics cooperating with the surface reaction. Finally, a rotating triboelectric nanogenerator was designed for environmental wind energy harvesting and driving the sensor, which provides a new solution idea for a new energy-integrated system strategy of agricultural modernization.

## Data Availability

Data are contained within the article.

## Supplementary Materials

The following supporting information can be downloaded at: https://www.mdpi.com/article/10.3390/s25092753/s1, Figure S1: XRD curves of prepared materials. Figure S2: XRD standard XRD curves of the products. (a) CuO (Reference code: 00-045-0937), (b) CeO_2_ (Reference code: 00-034-0394).
