# Supplementary material for: Gas-Sensing Study and Applications of Triboelectric Nanogenerator-Powered CuO-Modified CeO2 Nanomaterials for Ammonia Sensor at Room Temperature
Source: Sensors (Basel). 2025 Apr 26;25(9):2753. doi: 10.3390/s25092753 (PMC12074411; doi:10.3390/s25092753)
Supplement: Supplementary file 1 [file sensors-25-02753-s001.zip › sensors-3552851-supplementary.pdf]

Gas-sensing Study and Applications of TENG-powered CuO-Modified CeO2 Nanomaterials for Ammonia Sensor at Room Temperature

Junsheng Ding [1], Yingang Gui [2] and Hua Huang [2,*]

[1] College of Engineering and Technology, Southwest University, Chongqing 400715, China;djsdjs123@email.swu.edu.cn (J.D.); yinganggui@swu.edu.cn (Y.G.); huahuang@swu.edu.com (H.H.).
* Correspondence: huahuang@swu.edu.com.

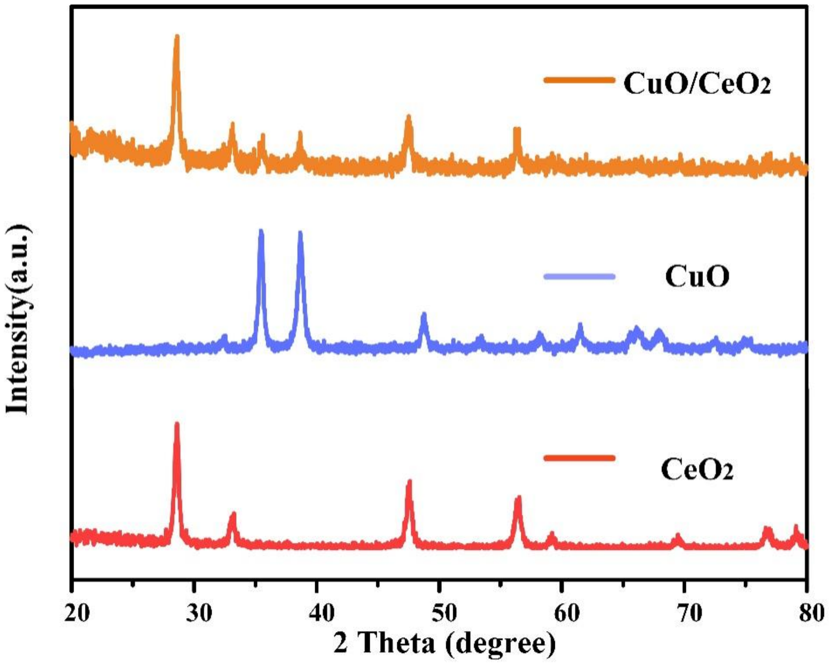

Figure S1 XRD curves of prepared materials

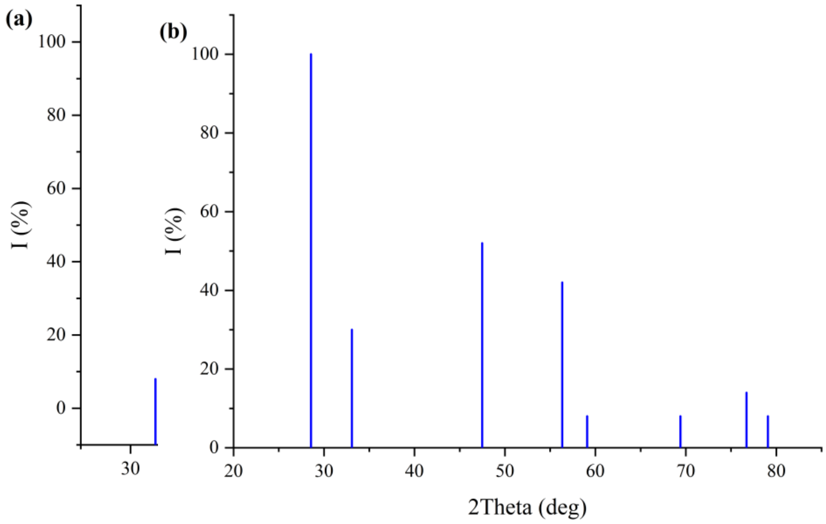

Figure S2 Standard XRD curves of the products. (a) CuO (Reference code: 00-045-0937), (b) CeO_2 (Reference code: 00-034-0394)

Reference code: 00-034-0394

Peak list of CeO_2

No.	h	k	l	d [A]	2Theta[deg]	I [%]
1	1	1	1	3.12344	28.555	100.0
2	2	0	0	2.70564	33.082	30.0
3	2	2	0	1.91341	47.479	52.0
4	3	1	1	1.63181	56.335	42.0
5	2	2	2	1.56221	59.087	8.0
6	4	0	0	1.35308	69.402	8.0
7	3	3	1	1.24149	76.700	14.0

8	4	2	0	1.21012	79.070	8.0

Reference code: 00-045-0937

Peak list of CuO

No.	h	k	l	d [A]	2Theta[deg]	I [%]
1	-1	1	0	2.75300	32.497	8.0
2	0	0	2	2.52700	35.496	100.0
3	1	1	1	2.32300	38.731	91.0
4	2	0	0	2.31000	38.958	28.0
5	-1	1	2	1.96140	46.249	2.0
6	-2	0	2	1.86730	48.727	20.0
7	1	1	2	1.77690	51.381	1.0
8	0	2	0	1.71280	53.453	6.0
9	2	0	2	1.58050	58.337	9.0
10	-1	1	3	1.50580	61.535	15.0
11	0	2	2	1.41840	65.787	8.0
12	-3	1	1	1.40960	66.250	11.0
13	1	1	3	1.37850	67.945	9.0
14	-2	2	0	1.37590	68.091	11.0
15	3	1	1	1.30380	72.429	5.0
16	0	0	4	1.26490	75.032	5.0
17	-2	2	2	1.26210	75.227	6.0
18	-2	0	4	1.19610	80.183	2.0
